# *Thismiasumatrana* (Thismiaceae), a new species from West Sumatra, Indonesia, with discussions on the taxonomic identity of *Thismiaclavigera*

**DOI:** 10.3897/phytokeys.113.29103

**Published:** 2018-12-06

**Authors:** Kenji Suetsugu, Hirokazu Tsukaya, Nurainas Nurainas, Hiroshi Okada

**Affiliations:** 1 Department of Biology, Graduate School of Science, Kobe University, 1-1 Rokkodai, Nada-ku, Kobe, 657-8501, Japan Kobe University Kobe Japan; 2 Department of Biological Sciences, Faculty of Science, The University of Tokyo, 7-3-1 Hongo, Bunkyo-ku, Tokyo 113-0033, Japan The University of Tokyo Tokyo Japan; 3 Bio-Next Project, Exploratory Research Center on Life and Living Systems, National Institutes of Natural Sciences, Yamate Build. #3, 5-1, Higashiyama, Myodaiji, Okazaki, Aichi 444-8787, Japan National Institutes of Natural Sciences Okazaki Japan; 4 Herbarium Universitas Andalas, The Department of Biology, Faculty of Mathematics and Sciences, Andalas University, Kampus Limau Manis Padang, 25163, West Sumatra, Indonesia Andalas University West Sumatra Indonesia; 5 Institute of Natural Environmental Sciences, University of Hyogo, Sanda, Hyogo 669-1546, Japan University of Hyogo Kyotanabe Japan; 6 Osaka City University, Sugimoto, Sumiyoshi-ku, Osaka 558-8585, Japan Osaka City University Osaka Japan

**Keywords:** *
Thismia
*, taxonomy, new species, mycoheterotrophy, Sumatra

## Abstract

A new species of the mycoheterotrophic genus *Thismia* Griff. (Thismiaceae), *Thismiasumatrana* Suetsugu & Tsukaya, from West Sumatra, Indonesia, is described, based on a rehydrated herbarium specimen from National Museum of Nature and Science, Japan. *Thismiasumatrana* is closely related to *T.clavigera* (Becc.) F.Muell. but is distinguished by a much larger flower.

## Introduction

*Thismia* Griff. is a genus of rare mycoheterotrophic plants that are mainly distributed in tropical Asia. Although only about 30 *Thismia* species were recognised until the 1990s, the diversity of *Thismia* is likely far underestimated, owing to the plants’ inconspicuous nature (i.e. highly reduced aboveground parts and small size that allows them to be easily covered by fallen leaves). Indeed, as more comprehensive field expeditions have been undertaken, a number of *Thismia* taxa have been discovered and described from various countries in Asia (Chantanaorrapint et al. 2016, Kumar et al. 2017, Sochor et al. 2017, 2018a,b, Suetsugu et al. 2017, 2018a,b, Sujanapal et al. 2017, Tsukaya et al. 2017, Chantanaorrapint and Suddee 2018, Hroneš et al. 2018, Nishioka et al. 2018, Yunoh 2018). Accordingly, the genus has been updated to include ca. 80 accepted species, making it one of the most species-rich mycoheterotrophic genera amongst vascular plants.

Based on these findings, it is likely that botanical surveys in tropical Asia will continue to uncover other previously undescribed taxa and present new distribution records. In addition, it is possible that the re-examination of herbarium specimens in natural history museums could reveal additional diversity (e.g. Suetsugu et al. 2018b). Here, *Thismiasumatrana* Suetsugu & Tsukaya, from West Sumatra, Indonesia, is described, based on the specimen from National Museum of Nature and Science, Japan (TNS), with the habitat information obtained by Nurainas Nurainas and Hiroshi Okada.

## Materials and methods

The examined specimen included two individuals (one in flower and one with immature fruit) that were mounted on a single sheet. As previous studies have suggested that the precise identification of *Thismia* specimens requires the observation of floral characteristics that are hidden in the perianth tube (e.g. Sochor et al. 2018a,b), the flowering individual of the specimen was removed from the herbarium sheet and rehydrated, by boiling in water for ca. 2 min, in order to investigate the specimen’s inner floral morphology. However, since the rehydration by boiling process was inadequate for dissection, the plant was kept in room temperature water for ca. 1 d. After that, the plant was carefully dissected and preserved in 70% ethanol. The fruiting plant remained dry and on the herbarium sheet. The structure of both individuals was observed using a stereomicroscope (MZ16a; Leica Microsystems, Cambridge, UK). It should be noted that our measurements, except for that of the inner floral parts, are based on the dry herbarium specimen and that the measurements may be smaller than those found in nature, owing to desiccation and shrinkage. In fact, during rehydration, the size of the inner floral parts increased by ca. 10% compared with that of the dry condition.

The morphological characters of both the unknown specimen and its putative closest relative, *T.clavigera*, were compared with detailed images of the holotype deposited in FI from JSTOR Global Plants (http://plants.jstor.org/) and previously published descriptions, illustrations and photographs of *T.clavigera* [i.e. Beccari (1878), Jonker (1938), Stone (1980), Chantanaorrapint and Chantanaorrapint (2009)]. The morphological characters of *T.clavigera* were evaluated based on the following criteria: (i) when the previous descriptions contained information on the lengths of the targeted parts, the values were used and (ii) when the descriptions did not contain information on the lengths of the targeted parts, the lengths were inferred from illustrations, photographs or images and the lengths of the parts that were included in the descriptions of Table 1 for comparison with the unknown specimen. Herbarium abbreviations follow Index Herbariorum (Thiers 2018, http://sweetgum.nybg.org/science/ih/).

## Taxonomy

### Thismia sumatrana

Taxon classificationPlantaeDioscorealesBurmanniaceae

Suetsugu & Tsukaya sp. nov.

urn:lsid:ipni.org:names:77192417-1

Figs 1 , 2

#### Diagnosis.

*Thismiasumatrana* differs from its close relative *T.clavigera*, in having a much larger flower (ca. 8 cm vs. ca. 2.8 cm long).

#### Type.

INDONESIA. West Sumatra: Padang Pariaman, Sipisang, ca. 300 m alt., 0°33'S, 100°21'E, 27 Feb 1994, *Okada et al. 112* (holotype TNS!, dried plant on a herbarium sheet (TNS-01051838) and liquid-preserved material in a bottle, labelled as the same specimen).

Terrestrial, mycoheterotrophic herb. Roots not seen. Stems erect, unbranched, 5–7.5 cm long. Leaves ca. 10, scale-like, appressed, triangular-ovate to lanceolate, 3–9 mm long, apex acute or slightly acuminate. Flowers solitary, sessile, terminal. Floral bracts ovate-lanceolate, 10–13 mm long, apex acute to acuminate, slightly curved. Flowers bisexual, ca. 8 cm long (including appendages); perianth tube, campanulate, ca. 2.7 by 1.3 cm, narrowest just above the ovary, widest at the top, with 12 longitudinal ribs, transverse bars inside present; outer perianth lobes 3, broadly triangular, ca 2.5 mm long; inner perianth lobes 3, incurved, apically adnate to form a dome-shaped mitre with three lateral holes, dome-shaped mitre ca. 9 mm in diam., bearing three eaves-like to hood-like accessory lobes at the tip and three slender claviform appendages at the top, appendages ca. 3.8 cm long; stamens 6, borne on the thickened margin of the perianth tube; filaments short, ribbon-shaped, free; connective broad, connate to form a tube with a quadrangular lateral appendage, apex acute, hairy; individual connective bearing four thecae; theca oblong, 1.2–1.4 mm long on the uppermost part of connective; interstaminal gland rectangular, 1.2–1.4 mm long on the line of fusion between each connective; style short, ca. 0.8 mm long; stigmas elliptic-oblong, ca. 2.5 mm long, 3-lobed; apex of lobes truncate; ovary ca. 5 mm long, cup-shaped. Mature fruit and seeds not seen.

**Figure 1.**
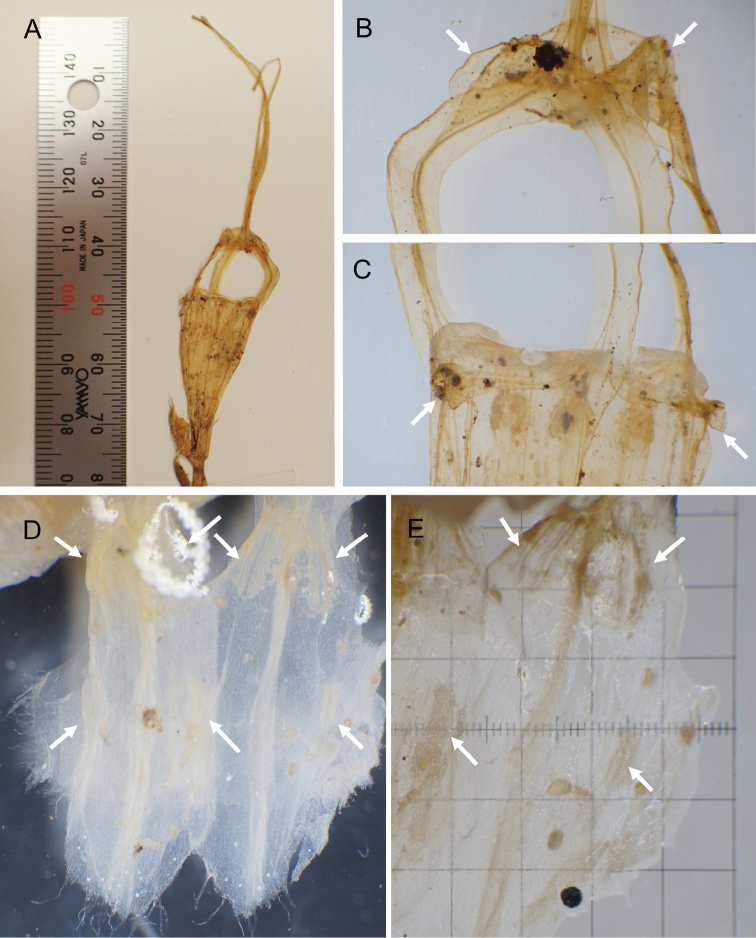
*Thismiasumatrana* from West Sumatra, Indonesia (from the holotype). **A** Flower **B** Dome-shaped mitre bearing the eaves-like to hood-like accessory lobes. The accessory lobes are indicated using the arrows **C** Upper part of perianth tube. Outer perianth lobes are indicated by arrows **D** Inner view of two pendulous stamens. Thecae and glands are indicated by upper and lower arrows, respectively **E** Outer view of a pendulous stamen. Thecae and glands are indicated by upper and lower arrows, respectively. One grid is equal to 1 mm^2^.

#### Distribution.

It is known from only a single collection comprising of one flowering and one fruiting individual.

*Thismiasumatrana* was collected from a forest floor beside a rheophytic zone along Anak Air Ganggu (Ganggu Stream), 0°33'S, 100°21'E, at Sipisang Village, Padang Pariaman, West Sumatra, Indonesia. The area was covered by mixed primary and secondary forest along a stream, where relatively natural conditions remained. For example, there were many individuals of a rare and large herbaceous plant, *Amorphophallustitanum* (Becc.) Becc. (Araceae). In addition, many individuals of a rheophytic plant, *Furtadoasumatrensis* M.Hotta (Araceae), grew on small rocks both in the stream and on the stream bank (Mori and Okada 2001).

**Figure 2.**
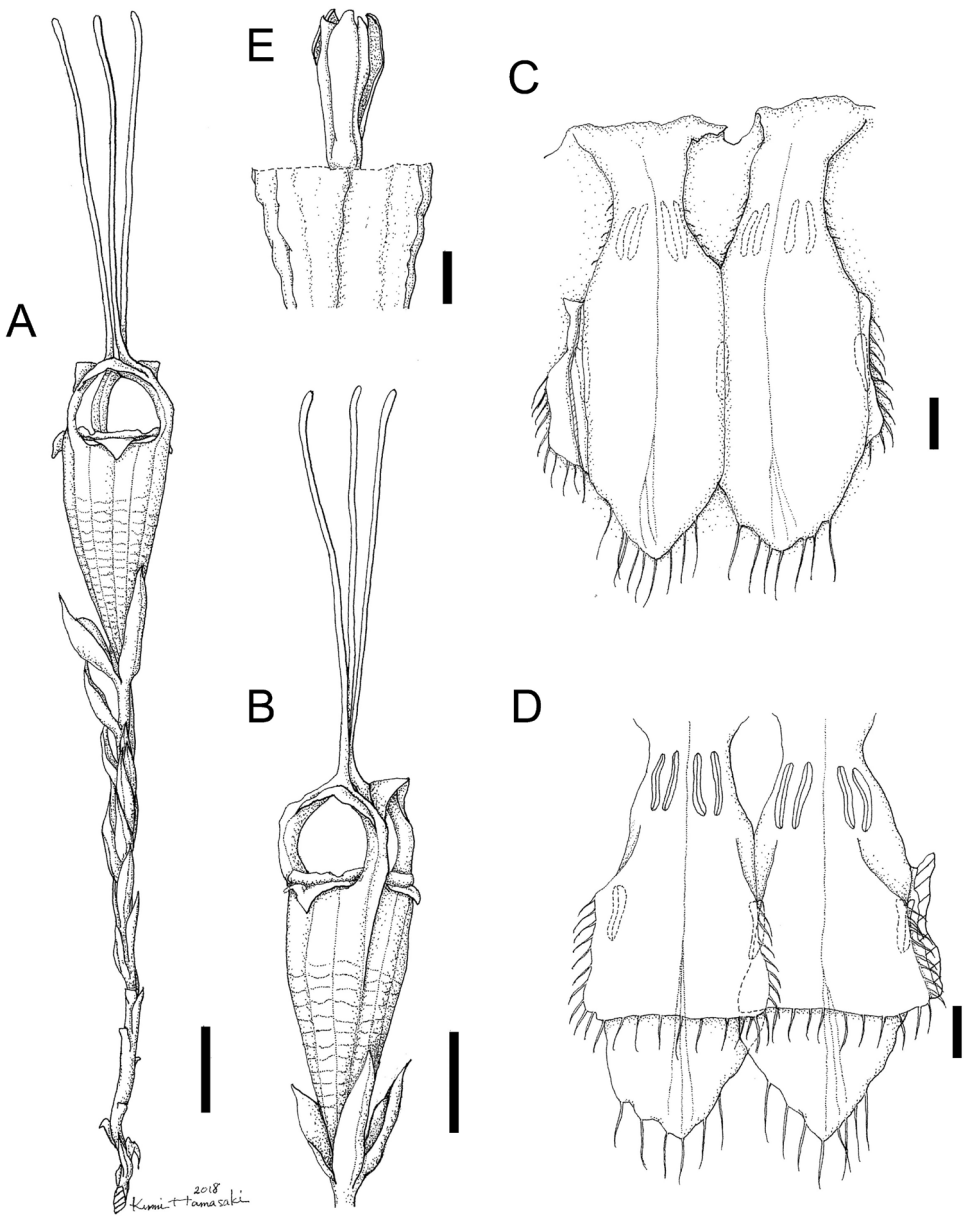
*Thismiasumatrana* from West Sumatra, Indonesia (Drawn from the holotype). **A** Flowering plant **B** Flower **C** Inner view of two pendulous stamens **D** Outer view of two pendulous stamens **E** Ovary with style and stigmas. Drawn by Kumi Hamasaki. Scale bars: 1 cm (**A–B**), 1 mm (**C–E**).

#### Taxonomic notes.

The distinctive characteristics of *Thismiasumatrana* include 1) minute outer tepals, 2) stamens with acute distal parts and 3) large flower. The combination of the first two characteristics, which have also been reported for *Thismiaclavigera* and *T.kelantanensis*, but not for the other *Thismia* species (Stone 1980, Chantanaorrapint and Chantanaorrapint 2009, Tsukaya and Okada 2012, Yunoh 2018), suggests that *T.kelantanensis*, *T.sumatrana*, and *T.clavigera* are closely related. However, *T.kelantanensis* can be easily distinguished from the other two species by the six-partite hood on its mitre (Yunoh 2018).

*Thismiaclavigera* was originally described as a member of the genus *Geomitra* Becc., based on collections from Sarawak in the early 1860s (Beccari 1878). Unfortunately, Beccari (1878) did not describe the inner floral characteristics, such as the structure of the connectives that are crucial in the identification of *Thismia* species and, instead, provided detailed drawings of the taxon’s external appearance. Nonetheless, comparing our material to Beccari’s original description, illustration and holotype specimen of *T.clavigera* revealed that *T.sumatrana* can be easily distinguished from *T.clavigera* by its much larger flowers (ca. 8 cm vs. ca. 2.8 cm long), whereas stems of *T.sumatrana* and *T.clavigera* are similar in length (ca. 5–7.5 cm long vs. 6–9 cm long; Table 1). As noted above, the flower of the *T.sumatrana* specimen may be shorter than those in nature, owing to desiccation and shrinkage. Nevertheless, the flowers are much larger than those of *T.clavigera*.

It should be noted that *T.clavigera* has been reported not only in type collections but also from different localities. Stone (1980) reported the rediscovery of *T.clavigera* from Pulau Langkawi, in the western part of the Malay Peninsula and Aceh, in northern Sumatra in 1979. Chantanaorrapint and Chantanaorrapint (2009) also reported that *T.clavigera* occurs on Tarutao Island, southern Thailand, which is close to Langkawi. In addition, one specimen seems to have been collected in Sarawak by Caddick (Caddick et al. 1998) and subjected to DNA sequencing by other authors (e.g. Merckx et al. 2006), although neither the precise locality nor the description is known, and no such specimen was found in K from where Caddick et al. (1998) reported on the deposited voucher specimen (Alison Moore, Curator of K, personal communication). However, we found that there are notable morphological variations amongst specimens recorded as *T.clavigera*.

The shape of the mitre, for example, varies amongst the specimens recorded as *T.clavigera* from different localities (Table 1). While Beccari (1877) depicted *T.clavigera* with small eave-like projections, such projections being very poorly developed in *T.clavigera* specimens from Langkawi and Tarutao (Stone 1980, Chantanaorrapint and Chantanaorrapint 2009) and the slender claviform appendages of the mitriform inner tepals are much shorter in *T.clavigera* from Aceh (ca. 5 mm) than in *T.clavigera* from other localities. Moreover, Stone (1980) also noted that *T.clavigera* from Aceh exhibits unique purple claviform appendages. However, these differences may only represent intraspecific variation, especially considering that Sochor et al. (2018b) noted that variation amongst mitre morphology is not necessarily taxonomically informative, at least for some *Thismia* species.

Yet, variations amongst *T.clavigera* from different localities have also been reported for other morphological characters. For example, *T.clavigera* specimens from Thailand exhibit orbicular interstaminal glands (Chantanaorrapint and Chantanaorrapint 2009), whereas the Langkawi specimens exhibit long, rectangular glands (Stone 1980) and the thecae of *T.clavigera* specimens from Thailand are situated slightly above the middle of the connective (Chantanaorrapint and Chantanaorrapint 2009), whereas those of Langkawi specimens are located in the uppermost part of the connective (Stone 1980). Therefore, considering that inner floral morphology is considered important for *Thismia* classification, differences in *T.clavigera* plants from Langkawi and Tarutao strongly suggest that at least two interspecific groups exist. Here, taxonomic treatments of specimens recorded as *T.clavigera* from other localities were not conducted because the inner floral characteristics of *T.clavigera* from the type locality are unavailable. Further investigation, based on specimens from the type locality of *T.clavigera*, is critical for elucidating the true taxonomic identities of specimens recorded as *T.clavigera* from other localities.

**Table 1.** Morphological comparison between *Thismiasumatrana* and the plants reported as *Thismiaclavigera*.

Characters	* T. sumatrana *	*T.clavigera* (type locality)^1^	*T.clavigera* (Langkawi)^2^	*T.clavigera* (Aceh)^2^	*T.clavigera* (Tarutao)^3^
Stem height	5–7.5 cm	6–9 cm	up to 12 cm	similar to Langkawi	up to 9 cm
Floral bract	10–13 mm long	6–7 mm long	up to 12 mm long	similar to Langkawi	ca. 12 mm long
Number of flowers	1	3	1–6	similar to Langkawi	1–2(–3)
Appendices on the tip of mitre	eaves-like to hood-shaped	eaves-like	poorly developed	similar to Langkawi	poorly developed
Length of perianth tube	ca. 27 mm	ca. 9 mm	ca. 13 mm	similar to Langkawi	15–19 mm
Length of claviform appendages	ca. 38 mm	8–12 mm	12–14 mm	ca. 5 mm long	19–32 mm
Size of outer perianth lobe	ca. 2.5 mm	ca. 1 mm	0.3–0.4 mm	similar to Langkawi	ca. 1 mm
Status of nectariferous gland on the top of mitre	rectangular, 1.2–1.4 mm long	unknown	rectangular, ca. 0.9 mm long	similar to Langkawi	orbicular, ca. 0.8 mm diameter
Status of thecae	oblong, 1.2–1.4 mm long, located in the uppermost part of connective	unknown	oblong, ca. 1 mm long, located in the uppermost part connective	similar to Langkawi	oblong, ca. 2 mm long, located little above the middle of connective

^1^from Beccari (1878), Jonker (1938) and our own examination of holotype image. ^2^from Stone (1980). ^3^from Chantanaorrapint and Chantanaorrapint (2009).

## Supplementary Material

XML Treatment for Thismia sumatrana
